# Paring and Suture Clamp Forceps

**Published:** 1889-04

**Authors:** Edmond V. Joye

**Affiliations:** Atlanta, Ga.


### PARING AND SUTURE CLAMP FORCEPS.*

BY EDMOND V. JOYE, M. D., ATLANTA, GA.

Being present, some years ago, at an operation for simple vesico-vaginal fistula, I noticed a great deal of time was consumed in taking up the vaginal mucous membrane, with the barbed hook, or tissue forceps, for the purpose of paring the edges of the fistula. Thinking this could be overcome by an instrument which combined the hook and elevator, thus raising the tissue and holding it *in situ*, I accordingly caused to be made the following instrument, which seems to meet all indications, and renders the operation comparatively simple and easy, reducing the time of its performance more than two-thirds. The instrument consists of two blades, each eleven (11) inches long, which are crossed and locked similarly to the "Hodge Forceps." Two inches of the extremities of the blades are made round, into which are inserted five (5) hooks equi-distant. The hooks are sharp and small at the free end, and thick where they enter the blade, (so made to avoid tearing out easily.) Shanks four (4) inches long, containing the screw lock of the "Hodge Forceps." The handles are five (5) inches long, slightly curved downwards, (in order to be out of the way of operator,) with rachet to hold and fix the blades

*Read before the Atlanta Society of Medicine, January 5, 1889.

in any desired position, either extended or brought together. The whole instrument is made as light as consistent with strength and durability.

The operation for simple vesico-vaginal fistula is thus performed: The patient is placed in the position recommended by Dr. J. Marion Sim's for such operation, viz: "Lying on the left side, with the thighs drawn up, or flexed, the right one a little higher than the left. Left arm thrown behind across the back, and the chest rotated forward, bringing the sternum nearly in contact with the table, while the spine is fully extended, with the head resting on left parietal bone." Chloroform is administered, Dr. Sims' duck-bill speculum introduced, an assistant holds up the right side of the nater.

The instrument being unlocked, the left hand blade is passed in the vagina, and hooked into the mucous tissue on the *right side* of the fistula, (not too near the edge,) and rotated from *left* to *right*, thus bringing the lock up with the points of the hooks looking toward the *right side;* and as the screw of the lock is upon the top of the blade, the handle is held down and the right hand blade is then passed in over the other, and the hooks hooked into the *left side* of the fistula, rotated from *right* to *left* and brought above and across the left hand blade. The blades are now locked, the handles brought together and fixed by the rachet. The fistula is thus firmly held, with its edges everted. The next step is to pare the edges, which is best done with Dr. Emmet's straight and curved scissors. This over, pass the needles, armed with silver wire, one in front of first hook, then one between each hook, and lastly, one on the outside of the last hook. Twist up the sutures and cut them off; unlock the blades, rotate them in the opposite direction from that when passed in, and hooked, thus relieving the hooks, withdraw the blades, and the operation is complete.

The advantages gained are these: 1st. By fixing the fistula with the instrument you are enabled to pare the edges at one stroke of the scissors, whereas, with the barbed hook or tissue forceps, you are compelled to do a great deal of nicking. 2nd. The blades constricting the pared edges of the fistula, furnish considerable support for the passage of the needle, which can be pushed through both sides at once, taking good hold at the same time, and also from this fact the sutures can be better twisted and adjusted. Lastly, the operator has entire control of the parts, for by depressing the handles he elevates the fistula, and during the paring and suturing can bring it clearly into view.

This simple instrument can be used in all operations wherein the principles of eversion, elevation and continuous and perfect adjustment of tissue is necessary, as laceration of perineum, colporrhaphy (anterior and posterior), elytrorrhaphy, etc. When performing the operation of elytrorrhaphy, the patient is placed in Sims' position, his duck-bill speculum introduced. The operator, determining his line of operation, passes in the *left hand blade* of the "paring and suture clamp forceps," and hooks it into the anterior wall of vagina, on the *right side* of the median line, and *rotates* the blade from *left* to *right.* He then passes in the *right hand blade*, over the one already in position, and hooks it into the anterior wall of vagina, on the *left side*, at same distance from the median line; *rotates* the blade from *right* to *left.* The right hand blade is then brought down upon and across the left hand blade, and locked. The operator can now put the tissue between the blades upon the stretch, by separating the handles, and holding them extended by the ratchet. He can then bring the mucous membrane so stretched clearly into view, by depressing the handles, and is thus enabled to readily denude or freshen the mucous membrane in any given direction, such as Sims' V-shaped operation for procidentia, apex to vulva, base to os uteri; or Emmet's operation for procidentia, the three points, one in front of os uteri, and the two lateral ones are easily located, and freshened while the tissue is firmly held by the forceps; or, as is suggested by Mundè, all the intervening tissue between the blades can be freshened or denuded, and the sutures passed and twisted up. The operator having entire control of the parts, by elevating or depressing the handles of the forceps, and the blades giving support to the tissue, allows him to pass in the needles, armed with sutures, more accurately. The operation is completed by drawing the handles of the forceps together, thus bringing the freshened surface into accurate and perfect contact, without any traction upon the sutures whatsoever. The sutures are then drawn up, twisted and cut off, the blades unlocked, and *each rotated* in the opposite direction from that when applied, unhooked and withdrawn. Emmet's, Mundè's and Stoltz' operations for cystocele can be greatly facilitated by the use of this instrument; also like operations on the posterior vaginal wall. Emmet's operation for urethrocele is thus performed with the "paring and suture clamp forceps:" Sims' speculum is introduced; the *left hand* blade is passed into the vagina and hooked into the anterior vaginal wall, on the *right* side, parallel to and one inch and a half from the point where the *slit in the urethra* is to be made, and *rotated* from *left* to *right*, and held in this position. The *right hand* blade is then passed in and hooked into the anterior vaginal wall, on the *left side*, at a point immediately opposite to the other blade, and at same distance from the median line, and rotated from right to left, bringing it down to and across the left hand blade, the blades are locked, and the handle separated until the tissue between the blades is placed upon the stretch; the handles are then fixed by the ratchet, a grooved sound is passed into the urethra, and the *slit* cut just in front of the neck of the bladder. "The hyperplastic mucous urethral membrane is drawn through this *slit* with a tenaculum, trimmed down until the urethral canal appears free, and its border is sewed by fine silk or catgut sutures to the mucous membrane of the vagina." Should the operator desire to constrict the edges of the *slit*, he can unlock the blades and unhook them, and rehook them on either side of, and near the *slit*, rotating them as before, and hold the edges firmly everted, while stitching the urethral mucous membrane to the mucous membrane of the vagina. He then unlocks the blades, and rotating them in opposite direction from that when applied, withdraws them. Cystocele in many cases can be overcome by paring the tissue and making two longitudinal wounds in the anterior wall of the vagina, parallel to and on either side of the urethra, extending from either side of the os uteri to the edges of the vulva. These, when sutured and healed, will form two longitudinal cicatrices, which will give support to the relaxed anterior wall. The posterior wall can be so treated for rectocele, the wounds to extend from either side of the posterior vaginal pouch to the edges of the vulva, parallel to, and on either side of, the median line. In procidentia, the paring and suturing of both the anterior and posterior walls in the directions mentioned, so as to form, when healed, four longitudinal cicatrices, which will act as four splints, preventing the sinking down of the uterus, or the shortening of the antero-posterior diameter of the vagina. The width of the denuded surfaces in any of the operations must be determined by the condition of the case. The paring and suturing can be done very readily with the "paring and suture clamp forceps," manipulated as before described. In perineorrhaphy, Mundé says: "While denudation is under process the index and middle finger of the left hand of the assistant who holds the left leg, and the right hand of the other assistant, separates the labia by traction on the sound skin, and enables the operator to see the vaginal canal as he proceeds inwards, this traction must be uniform and is regulated by the operator at will during the whole operation, so as to secure the best possible symmetry of the two halves of the womb, and exactly corresponding points of entrance and exit of the sutures." This traction can be made with this instrument with more uniformity than with the hands of an assistant, and the instrument gives the operator complete control of the parts, both when denuding and suturing. The instrument is applied in this manner: The *left hand* blade is hooked into sound skin on the right side of the lacerated perineum, rotated from *left to right*, then the *right hand* blade is hooked into sound skin on the left side of the wound, and rotated form *right to left*, the blades are then locked. Now by separating the handles, fixing them so separated, by the ratchet, the operator can by depressing the handles draw the sides of the wound tense, and thus causing the wound to gape at its outer edges, readily bring the tissue at the bottom of the rent to view, the tense condition of the sides allows the operator to pare them with Emmett's flat scissors, kept level with the surface, without any danger to the suctures below the mucous membrane. The paring done, the sutures are passed; after this, the wound and vagina are thoroughly cleansed, the handles of the instrument are brought together, which causes the blades to bring the two halves of the freshened wound into close contact and firmly hold them in this position. The operator draws up the sutures one by one and twists them, unlocks the blades and removes the instrument. *There is no traction upon the sutures to bring the edges of the wound together.* The edges of the wound are held together by the blades of the instrument, and by so supporting them, enables the operator to twist up, and tighten the sutures with more uniformity, than he can possibly do by drawing the wound together by the sutures. Again, when the blades are removed, *the strain comes alike upon all the sutures at one and the same time*, and greatly diminishes the danger of their tearing out.

One blade of the instrument can be used when the two are not thought necessary. I have had the hook-shank so made that additional hooks can be screwed into the ends of the blades, making the hook-shank longer, and capable of embracing more tissue, if such is desired.

